# Special issue on the human microbiome: from symbiosis to therapy

**DOI:** 10.1038/s12276-020-00488-5

**Published:** 2020-09-10

**Authors:** Amit Sharma, Sin-Hyeog Im

**Affiliations:** 1 Department of Life Sciences, Division of Integrative Biosciences and Biotechnology, Pohang University of Science and Technology, Pohang, Gyeongbuk 37673 Republic of Korea; 2 ImmunoBiome Inc. POSTECH Biotech Center, Pohang, 37673 Republic of Korea

**Keywords:** Experimental models of disease, Translational research

The symbiotic microbes harbored at various sites in the human body are increasingly being appreciated as a dynamic extension of the human host. Earlier investigations appreciated the coevolution of the microbiome with the host, particularly that associated with the gut, to achieve metabolic efficiency for the host and nutrient provision for the microbes. However, the identification of host–microbiome interactions at various physiological and immunological levels has provided a stimulus to the field, wherein commensals are increasingly being explored for therapeutic benefits. There is now ample evidence that links perturbations in microbial communities to a range of pathologies, such as inflammatory bowel disease^1^; autoimmunity and allergic disorders^2^; cardiovascular disorders^3^; neurologic diseases, including autism spectrum disorders^4^, Alzheimer disorders^5^, Parkinson’s disease^6^, and depression^7^; and cancers^8,9^. In the fetomaternal case, it is now well established that babies receive their initial dose of microbes from mothers during passage through the birth canal and other body surfaces^10^. Vaginal microbes can also be used in risk assessment for preterm births^11^.

The National Institute of Health Human Microbiome Project (HMP) and the integrated HMP have explored the existence of a universal “healthy human microbiome” and host–microbiome interplay regarding immunity, metabolism, and molecular activities, respectively^12^. These data have enabled researchers and drug developers to look for not only living microbial therapeutics but also small molecule therapeutics related to the human microbiome^13^.

In this special issue titled “Human microbiome: from symbiosis to therapy”, four insightful reviews discuss the criticality of microbiome research from a perspective of their immune interactions with the host, critique of preclinical as well as clinical research, and manufacturing regulations and guidelines.

In the first review, Beukema and colleagues^14^ discussed the impact of dietary fibers on shaping the gut microbiome. As is evident from various reports, dietary fibers can be converted into fermentation products, such as short-chain fatty acids, and other metabolites that play essential roles in shaping the gut immune system. This review provides an extensive deliberation of α-1,4-linked galacturonic acid-containing heteropolysaccharides and pectins. These compounds are found in the cell walls of various fruits and vegetables. Based on their physicochemical properties, pectins can have varied effects on the gut environment and the host immune system. Bacterial species that possess carbohydrate-active enzymes can break down pectins and provide them as nutrients to other microbes, effectively influencing the gut microbiome. On the other hand, fermentation of pectins can boost the production of short-chain fatty acids, which in turn can have far-reaching effects on the gut immune system. Pectins have also been reported to suppress the pathogen load in the gut by blocking their adhesion to gut cells. The authors emphasize that it is important to perform additional controlled human studies to ascertain the effect of pectins, as humans possess a diverse microbiome. In addition, caution needs to be applied in using dietary fibers for inflammatory bowel disease, as the diseased gut can lead to faulty fermentation of dietary fibers, with subsequent undesirable consequences.

Furthermore, in addition to diet, alimentary system bacteria also shape the local immune system. Ohno et al.^15^ described bacterial diversity in the human stomach. The stomach microbiota, in particular, *Helicobacter pylori*, shapes the stomach immune system vis-a-vis the local group 2 innate lymphoid cell (ILC2) population. ILC2s are dominant ILCs in the stomach and are dependent on the microbiota for their existence, unlike their counterparts in other tissues. Stomach ILC2s are highly responsive to IL-7-induced proliferation, whose production is instigated by the stomach microbiota. ILC2s, in turn, help in the production of IgA by secreting IL-5, which modulates stomach microbial composition. A persistent *H. pylori* infection is acquired in childhood, while infections in adulthood are easily cleared after an episode of acute gastritis. Most likely, the presence of an immature and less developed ILC2 population in childhood is the reason for this phenomenon. Thus, there is an interdependent modulation of the gastric immune system and microbiota.

To explore the functional importance of the human microbiome in health and disease, the development and establishment of animal models are critically important. The humanized gut microbiome model can be instrumental for mechanistic deductions of microbial activities and effects and subsequent preclinical development of therapies. Mice derived from a germ-free status are a very important tool for colonization with a single bacterium or a simple defined mixture of bacteria for investigation of specific mechanisms. In addition, germ-free mice are essential for phenotype transfer studies using either whole human feces or a complex mixture of defined human microbes. However, there can be several confounding factors in the development of these models. Park et al.^16^, in a very comprehensive review, deduced various factors and conditions that can affect the development of and results from such animal models. Apart from the obvious differences in anatomy, physiology, and gut microbiota composition between humans and mice, there are several other factors that should be duly considered. For the purpose of screening, the generation of a stock human gut microbiota transfer model can be seriously affected by the provenance of fecal samples for reconstitution of the microbiota in mice. Among other population-level factors, human fecal microbial composition and diversity are greatly affected by ethnicity, geography, and nutritional as well as socioeconomic status. At the individual level, the donor’s diet, physical activity, etc. are the factors to be considered before fecal microbiota transfer into germ-free mice. Although generalization is extremely difficult, as every individual possesses their unique microbiota “signature”, such considerations will help in bringing uniformity in model generation. Several experimenter-originated confounding factors are also discussed that can affect the outcomes of such a model. These include the preparation of fecal samples and the route of administration to the animals. The authors describe how fresh fecal samples are superior to frozen ones and a rectal delivery better mimics the clinical setting while reducing the occurrence of sepsis in recipient mice. Furthermore, prevalent husbandry practices can significantly alter the reconstitution of the human gut microbiota. Although no preclinical model can absolutely mimic human pathophysiology, various approaches, such as human immune cell reconstitution in immunodeficient germ-free animals along with human gut microbiota and an optimal diet to maintain the initial microbiome status, can be utilized. Such animals can be further genetically humanized for human epithelial colonization factors and human cytokines, resulting in better microbial colonization and greater immune cell engraftment, respectively.

Probiotics as a food supplement or nutraceutical are naturally meant to be used in a healthy population. Owing to the long-term consumption of various food-derived commensals, they are considered safe. However, the concern for safety and toxicity of such preparations increases manyfold once they are intended for use as a prophylactic or therapeutic “drug” in vulnerable subjects. In the final article of this series, Cordaillat-Simmons et al.^17^ systematically discussed various aspects of the regulatory framework suggested by the “International Council for Harmonization of Technical Requirements for Pharmaceuticals for Human Use”. They propose several recommendations for developers of living biotherapeutic products (LBPs) so that they can successfully navigate their products through the clinical approval process. All LBPs must demonstrate a positive benefit-risk balance by ensuring the quality, safety, and efficacy of the products. For microbial therapeutics, quality must be ensured during the manufacturing process, which includes upscaling and batch-to-batch manufacturing. In addition, demonstration of stability both in terms of potency and genetic stability for the intended duration of storage is mandated. The authors discuss, in detail, the identification of critical parameters that can influence these factors. Early establishment of potency testing based on the mechanism of action can help in controlling the quality of the product during its evolution and upscaling. Furthermore, affirmation of safety for LBPs is different from chemical drug development processes, as once in the gut, microbes can produce metabolites that can affect host functions and present unintended consequences, expanding the scope of toxicological screenings. This necessitates that the safety of these products should be ensured from the very start of the development process. Unlike for new chemical entities, there are no established GLP safety assays for LBPs; thus, the authors suggest employing a spectrum of preclinical research models to generate a global safety profile of the product for the intended target population. Similarly, there are practical difficulties with demonstrating the efficacy of LBPs in clinical settings, as various environmental and host-related factors can affect the outcome. The authors also suggest designing “bridging” trials for extrapolation of data from one region to another if the LBP is being developed for international usage.

Overall, these four articles in this special issue provide an interesting overview of the interaction of humans and their microbiome to develop microbiome therapeutics. The overarching outlook of a complete therapeutic LBP development process provides a singular resource. As an increasing number of reports suggesting the role of the microbiome in health and disease are coming out, a unique opportunity to utilize the microbiome for the benefit of human health presents itself. However, the field is still nascent, and due caution is warranted in establishing preclinical research methods as well as clinical research guidelines. In addition, a reductionist approach would be helpful to identify and isolate effector molecules (active pharmaceutical ingredients) from LBPs and elucidate their mechanisms of action. It remains to be seen if such derivatives can be developed like normal small molecule drugs and/or biologicals.
